# Could *α*-Synuclein Amyloid-Like Aggregates Trigger a Prionic Neuronal Invasion?

**DOI:** 10.1155/2015/172018

**Published:** 2015-03-19

**Authors:** Maria Antònia Busquets, Alba Espargaró, Joan Estelrich, Raimon Sabate

**Affiliations:** Department of Physical Chemistry, Faculty of Pharmacy, University of Barcelona, and Institute of Nanoscience and Nanotechnology of the University of Barcelona (IN2UB), Avenue Joan XXIII 27-31, Barcelona, 08028 Catalonia, Spain

## Abstract

Parkinson's disease (PD), a progressive neurodegenerative disease primarily affecting voluntary and controlled movement, is characterized by abnormal accumulations of *α*-synuclein (*α*-syn) in intraneuronal Lewy bodies. In the last years, the increased number of evidences from both the* in vitro* and* in vivo* studies has shown the ability of *α*-syn to misfold in amyloid conformations and to spread via neuron-to-neuron transmission, suggesting a prion-like behaviour. However, in contrast to prion protein (PrP), *α*-syn transmission is far from neuronal invasion. The high neuronal toxicity of both mature fibres and oligomeric species, as well as the intracellular localization of the protein and the difficulty to be secreted, could be key factors impeding the prion ability of *α*-syn aggregates.

## 1. Introduction

Parkinson's disease (PD), the second most common neurodegenerative illness after Alzheimer's disease (AD), is a progressive debilitating degenerative disease, primarily affecting voluntary and controlled movement, characterized by dopaminergic neuron loss in the motor regions [1, 2]. It is widely accepted that neuronal death and associated pathology of PD are related to the formation of filamentous intracellular aggregates termed Lewy bodies [2]. The main component of these aggregates is *α*-syn, a presynaptic neuronal protein of 140 amino acids encoded by a gene on chromosome 4 with a putative role in synaptic function and neural plasticity [3]. In solution, *α*-syn is considered to be an intrinsically disordered protein. However, soluble monomers may occasionally self-polymerize into amyloid structures under a nucleation-elongation process [4].

Amyloid aggregates, characterized by displaying a core region formed of repetitive arrays of *β*-sheets oriented parallel to the fibril axis [5, 6], are the hallmark of increased number of human diseases ranking from AD to Creutzfeldt-Jakob disease (CJD) [7]. In PD, *α*-syn self-assembly in amyloid-like structures, entailing the formation of Lewy bodies in brain, is directly related to symptomatology and neuronal alteration. Lewy bodies, initially located in the* substantia nigra* in the mesencephalon, are spread throughout the brain in the course of the disease appearing in several areas of the brain. As recently proposed, this spreading process could be caused by neuron-to-neuron spreading of *α*-syn amyloid species via axonal transport between connected areas [8]. Thus, *α*-syn has shown prion capacity in both experimental (in animal model) and natural (in humans) transmissions. In these cases, the transmission is cell-to-cell and host-to-graft, that is, by direct cellular contact [9]. However, *α*-syn transmission is far from neuronal invasion (process characterized by exponential multiplication in an appropriate ghost and transmission between individuals by various routes) as shown by prion protein (PrP). This dramatic difference should be explained for the limitations in the spreading of *α*-syn amyloid-like species, mainly due to the intrinsic toxicity of *α*-syn amyloid aggregates, low persistence of dispersible amyloid species, and localization of *α*-syn aggregates [10]. In consequence, *α*-syn aggregates seem unable of propagating long distance, although there is the possibility of aggregates being transmitted successively through multiple neuronal connections. This would involve the “secondary” secretion of seeded aggregates. Through this mechanism, *α*-syn aggregates could be spread over long distances.

## 2. *In Vitro* Evidences

*In vitro* studies have revealed that recombinant *α*-syn can polymerize from soluble unstructured monomer into amyloid *β*-sheet rich fibrils with morphologies and structural characteristics similar to those extracted from Lewy bodies of disease-affected brains [11]. Experimental aggregation conditions such as initial concentration, molecular crowding, temperature, pH, ionic strength, agitation, phosphorylation, and polyion presence can determine both aggregation kinetics (accelerating or inhibiting the fibrillation) and structural properties [11–14]. As observed in other amyloid aggregation processes, the formation of *α*-syn fibrils is a nucleation-dependent process that can be accelerated by the presence of preformed fibrils, which act as a template. This seeding behaviour, thought to promote the fast development of AD after its clinical detection and the infectivity of human prions, is a sequence specific process where aggregation is nucleated by homologous fibrils but not by fibrils from closely related sequences [7]. As observed in A*β* specific mutations, *α*-syn familial mutations could be responsible for the seeding of wild-type protein's fibrillation. Interestingly, it has been shown that the wild-type *α*-syn fibrils obtained in presence of preformed A30P aggregates display the same structural features of A30P seeds, denoting the template effect of preformed A30P fibrils [15]. *α*-syn mutations, associated with early onset familial disease (namely, A53T and A30P), polymerize more rapidly than wild-type* in vitro* [12]. This fact opens the possibility that spontaneous *α*-syn mutation in a cell, entailing accelerated amyloid aggregation, could trigger the *α*-syn ensemble in neighbouring cells as consequence of putative seeding processes via cell-to-cell transmission. In this case, since the specific mutation is only present in a small number of cells (if not in a single one), the fibrils of wild-type *α*-syn would be the unique checkable material derived from the chain reaction observable in neighbouring cells. Importantly, as in prion diseases, atypical wild-type fibrils and, consequently, different clinical symptomatology could be expected, which would imply the existence of different *α*-syn strains, as recently observed* in vivo* [16].

## 3. *In Vivo* Experimental and Natural Transmission Evidences

Recent studies have evidenced the* in vivo* transmission capacity of *α*-syn fibrils, showing the neuron-to-neuron transmission of exogenous *α*-syn amyloid-like aggregates in both cultured neuronal and nonneuronal cells and transgenic and wild-type mice [17–21]. The term “amyloid-like aggregates” refers to wide range of aggregate species, including fibrils, protofibrils, and oligomers. Any of these species presents the main properties specific of amyloids [6, 22]. In addition, these studies have also disclosed that both *α*-syn aggregates produced from synthetic/recombinant proteins and *α*-syn aggregates obtained from brains of patients or transgenic mice are capable of acting as polymerization seeds triggering the amyloid aggregation process [21]. Thus,* in vivo* *α*-syn fibrils are able to seed the polymerization of *α*-syn soluble monomers undergoing the formation of amyloid-like aggregates in cultured cells [20, 23, 24]. In the same way, *α*-syn aggregates from transgenic mice brains are capable of seeding and propagating the protein aggregation in the intracerebrally injected transgenic mice [25]. These observations have also been displayed in both transgenic and wild-type mouse brains, wherein the administration of preformed *α*-syn fibrils triggers the formation of Lewy bodies [17, 25–27]. Moreover, as shown in coculture models, human dopaminergic neuronal cells overexpressing *α*-syn (donor cells) are capable of transferring *α*-syn aggregates to neuronal cells without *α*-syn overexpression (acceptor cells), demonstrating the cell-to-cell transfer via releasing pathways [18]. Notably, it has also been stated that intracellular *α*-syn aggregates can be secreted to extracellular matrix and finally transferred to nearly neurons. Recent findings have suggested that *α*-syn aggregates could be transmitted from pathological neurons by different mechanisms [8, 18, 28–30].

More importantly, evidences of natural transmission of *α*-syn aggregates have been shown [31–35]. Postmortem studies of PD patients who have been transplanted years before with embryonic dopamine neurons into putamen reveal the presence of *α*-syn aggregates in these grafted regions. Since Lewy bodies are very unusual in young neurons, the presence of *α*-syn aggregates in transplanted neurons suggests an infection process from aged pathological neurons (donor cells) to young nonpathological neurons (acceptor cells) [31–35].

Although there is no strong evidence so far that amyloid aggregates are secreted (only one report shows that some of the secreted *α*-syn oligomers have *β*-sheet rich conformation [36]), the intracellular *α*-syn amyloid-like aggregates could be partially secreted to extracellular matrix wherein they could internalize via endocytosis into neighbouring neurons and act as templates/seeds that would trigger the *α*-syn self-aggregation process and would spread the pathology in the brain. Nevertheless, all reported transmission events are far from neuronal invasion, as observed in the case of prion protein (PrP), suggesting certain limitations on the putative spreading process of *α*-syn amyloid fibrils.

## 4. Limitations on ***α***-Syn Spreading

As recently proposed, the intrinsic toxicity of amyloid-like aggregates, the amount of small size amyloid aggregates (namely, oligomer and prefibrillar species), and the localization and putative secretion mechanisms could become key factors in the amyloid transmission and prion capability [10].

### 4.1. Intrinsic Toxicity of *α*-Syn Amyloid Aggregates

As indicated previously, the intrinsic cytotoxicity of each amyloid aggregate could become a key factor in the putative infectivity of amyloid species [10]. Seeding is essential in the infection, either by cell-to-cell transmission or by neuronal invasion. Thus, high contact times between external amyloid species and the walls of noninfected cells favour the amyloid penetration and internal accumulation of the seeds in the healthy cells, increasing the seeding process and the aggregation of soluble amyloid-prone protein in an exponential way. Thus, the toxicity of external amyloid aggregates could open two different scenarios. In the first, as observed in amyloid-*β* (A*β*) peptide, the contact between highly toxic aggregates and the wall of healthy cells would undergo membrane disruption, homeostasis alteration, and finally apoptosis and cell death [37–40]. The fast death of neighbouring cells, via membrane disruption, drastically reduces the contact time between external amyloids and internal soluble amyloid-prone protein, reducing the number of seeds in the healthy cell. Contrarily in the second scenario, the presence of amyloid aggregates of low toxicity would be linked to high infection capacity, as observed in prion protein (PrP) [41]. The low toxicity favours long contact times between the membranes of cells susceptible to be infected and the external amyloid aggregates, facilitating the penetration of an increased number of seeds as well as the transmission of the amyloid conformation.

It has been vastly shown that *α*-syn aggregates, from prefibrils and oligomers to mature fibrils, display remarkable cytotoxicity, both* in vitro* and* in vivo* [42–47]. Interestingly, it has been suggested that the toxicity of *α*-syn amyloid species is linked to membrane interaction wherein the presence of these species undergoes the pore formation and membrane disruption [4, 48–52]. These evidences suggest that *α*-syn amyloid aggregates display similar toxic properties that A*β* aggregates. In this way, in contrast to PrP, the high intrinsic toxicity of *α*-syn amyloid-like species could drastically reduce the spreading capacity of the protein, becoming a limiting factor in a putative neuronal invasion process. However, it is not clear that toxicity and infection capacity can be dissociated, and, in consequence, the fact that an increase of toxicity is related to a reduction of spreading capacity is, at the present, a hypothesis.

### 4.2. Amount of High Dispersible *α*-Syn Aggregates

For years, the mature fibres were considered the causative species of the toxicity in neurodegenerative processes as AD or PD. However, the increased number of evidences has unequivocally stated that fibril precursors such as oligomers and protofibrils are the primary origins of pathological behaviour [7]. It has also been shown that oligomers and low size species are the most dispersible and spreading amyloid material [41]. Interestingly, the oligomers usually display high intrinsic toxicity, and this feature could limit their dispersion capacity. Since the seeding capacity is directly related to the number of the seeds in the cell [53, 54], the concentration of oligomers could become a crucial factor in the amyloid propagation.

However, as the oligomer species are rather heterogeneous, there is a possibility that those species responsible for the toxicity are different from those that facilitate the transmission [55]. *α*-syn oligomeric species are usually detectable, both* in vitro* and* in vivo*, under a wide range of experimental conditions [56–60]. The presence of these transient species in the several phases of *α*-syn self-polymerization process as well as the high stability of some of them suggests that these species could be strongly implicated in the development of PD [56, 59, 61]. An increasing number of evidences shows that are *α*-syn oligomeric species, rather than mature fibrils, which display the highest toxicity, becoming the main responsible species of the *α*-syn pore capacity, dysfunction of calcium homeostasis, membrane disruption and finally neuronal death [11, 46, 62–65].

Remarkably, it has been stated that oligomeric *α*-syn species are not introduced into cells and do not act as seeds in the self-polymerization process in cultured cells [24]. However, although *α*-syn oligomers tend to induce membrane disruption and cell death, recent studies have shown that certain types of *α*-syn oligomers, produced* in vitro* under specific conditions, can be internalized by primary neuronal cells and neuronal cell lines, triggering the self-aggregation of soluble *α*-syn in healthy neurons [11, 55, 65].

In this context, we could speculate that though *α*-syn oligomers could be secreted and spread to the extracellular matrix, their extreme toxicity would provoke fast membrane alteration and cell death, with their penetration into healthy cells and putative seeding actions being useless. Consequently,* in vivo* models in which *α*-syn fibres, not oligomeric species, are capable of being cell-to-cell transmitted have been proposed [18, 66]. Thus, since amyloid fibres are poorly dispersible, the high membrane toxicity of oligomers could become a relevant handicap in a putative neuroinvasive process.

### 4.3. Location and Secretion Mechanisms of *α*-Syn Aggregates

As previously indicated, amyloid transmission and prion prevalence are directly related to the seeding capacity of each amyloid-like species of each amyloid-prone protein. In summary, this fact would imply that amyloid-prone proteins displaying species with high seeding capacity should show more ability to transfer the amyloid state, either neuron-to-neuron or real neuronal invasion. In this context, the localization of each amyloid-prone protein becomes essential for their transmission and spreading [10]. Thus, while extracellular proteins as A*β* or PrP would be good candidates for acting as prions, intracellular ones such as tau, ataxin, or *α*-syn would be bad candidates. It is of relevance to point out that recent studies have shown the dual localization, in extracellular and intracellular compartments, of an increasing number of proteins, including *α*-syn or tau [67–70]. This amazing fact opens the possibility that amyloid-prone proteins implied in high prevalent neurodegenerative diseases such as *α*-syn or tau, previously considered intracellular and hardly spreading ones, could display certain transmission and spreading properties.

At this point, essential differentiation between cell-to-cell transmission and distal-neuronal-spreading would be taken into consideration. Whereas cell-to-cell transmission implies a progressive infective process that could be completely insufficient to trigger massive neuronal invasion, distal-neuronal-spreading would be absolutely necessary for a putative massive neuroinvasion. Thus, the spreading of PrP^Sc^ along peripheral (spleen) and central nervous system (CNS) via distal-neuronal-spreading is termed neuroinvasion.

Interestingly, cell-to-cell transmission of a-syn amyloid-like aggregates could be carried out by release of aggregates from injured neurons to extracellular matrix via membrane damage of the host cell and then directly translocate into membrane of nearly neuron, transference via exocytosis and endocytosis mechanisms, accumulation into exosomes (or microvesicles) where the aggregates are secreted in a calcium-dependent manner and transmitted to neighbouring neurons, tunnelling nanotubes forming tubular membrane bridges interconnecting neurons, and direct synaptic contact [9, 11, 28, 35, 71, 72]. In contrast, distal neuronal-spreading should be limited to secretory process via exocytosis-endocytosis mechanisms.

Since *α*-syn can be considered as a cytoplasmatic protein, two putative limiting processes for distal spreading should be taken into account. On the one hand, there is the release of amyloid-like aggregates from injured cells to extracellular matrix, and on the other hand, there is the internalization of secreted amyloid aggregates into healthy cells. Significantly, *α*-syn amyloid aggregates have partially overcome these limitations. Thus, several forms of *α*-syn have been detected in extracellular biological fluids from the cerebrospinal fluid to human plasma and saliva [11, 69, 73, 74]. In addition, recent evidences have shown that *α*-syn, both monomers and amyloid-like aggregates, can be secreted by nonclassical vesicle-mediated exocytosis [75]. In the same direction, different pathways for the internalization of *α*-syn exogenous species have also been proposed. Thus, while *α*-syn monomers can pass across the membrane via passive transport, amyloid-like aggregates, namely, oligomers and fibres, penetrate into cells via endocytosis [11, 76]. This set of findings could open the possibility of putative distal-neuronal-spreading processes. However, as previously discussed, although either fibres or oligomers have been proposed as putative material to be propagated among cells, recent studies have shown that there are only mature fibres of *α*-syn, and not monomers and oligomers, responsible for triggering the amyloid aggregation process in healthy neurons, becoming the most effective seeds [18, 23, 66]. In summary, the fact that mature fibres, the less dynamic material, are the most effective seeding material suggests that whereas neuron-to-neuron transmission could be favoured, distal-neuronal-spreading is clearly disadvantageous.

## 5. Concluding Remarks

An increasing number of evidences suggest that *α*-syn shows certain prion capacity. However, although neuron-to-neuron transmission has been clearly demonstrated, massive neuronal invasion as consequence of fast distal-neuronal-spreading has not been observed. The intrinsic toxicity of *α*-syn fibres, peculiar characteristics of oligomeric species, and *α*-syn location could become key factors, determining *α*-syn prion ability. In *α*-syn case, the previously mentioned factors appear to act against a distal-neuronal-spreading, limiting the *α*-syn spreading to cell-to-cell transfection. In this way, *α*-syn oligomers, the most dispersible and dynamic structures, are not available to act as seeds, representing a clear impediment to distal-neuronal-spreading. Additionally, the high cytotoxicity shown for all *α*-syn amyloid-like aggregates, entailing membrane disruption and cell death, suggests also a handicap to *α*-syn spreading. Moreover, the neurotoxicity could be associated with seeded aggregation, within cells. In this case, toxicity and infectivity may not be dissociable. Finally, the fact that only certain amounts of *α*-syn can be detected in several biological fluids signifies that the cytoplasmic localization of the protein is another limiting factor for the distal protein spreading.
